# Supplementary material for: Visual attention is available at a task-relevant location rapidly after a saccade
Source: eLife. 2016 Nov 23;5:e18009. doi: 10.7554/eLife.18009 (PMC5120882; doi:10.7554/eLife.18009)
Supplement: Supplementary file 1. — (A) Experiments 1 and 2. False-positive rate from 0 to 150 ms after saccade offset shows no effect of post-saccadic retinotopic attentional persistence. (B) The false-positive rate in Experiment 3 shows no effect of either pre-saccadic predictive shifts or post-saccadic retinotopic persistence. DOI: http://dx.doi.org/10.7554/eLife.18009.017 [file elife-18009-supp1.docx]

**Supplementary File 1A: Experiments 1 and 2. False-positive rate from 0 to 150 ms after saccade offset shows no effect of post-saccadic retinotopic attentional persistence.**

|  | Distractor to right of target  (retinotopic persistence) | | Distractor below target  (control) | |
| --- | --- | --- | --- | --- |
|  | No. False-positives | Total trials | No. False-positives | Total trials |
| Experiment 1 | | | | |
| Subject BA | 0 | 16 | 1 | 9 |
| Subject JV | 0 | 25 | 0 | 12 |
| Subject JS | 0 | 18 | 0 | 15 |
| Subject JK | 0 | 17 | 0 | 17 |
| Subject KW | 1 | 27 | 3 | 21 |
| Subject LV | 0 | 26 | 0 | 23 |
| Subject MK | 0 | 10 | 0 | 12 |
| Subject SP | 4 | 24 | 1 | 18 |
| Total | 5 | 163 | 5 | 127 |
| Experiment 2 | | | | |
| Subject JV | 1 | 14 | 5 | 14 |
| Subject LV | 0 | 25 | 0 | 27 |
| Subject MK | 0 | 15 | 0 | 17 |
| Subject MS | 6 | 43 | 5 | 61 |
| Subject TY | 2 | 18 | 0 | 17 |
| Total | 9 | 115 | 10 | 136 |

**Supplementary File 1B: The false-positive rate in Experiment 3 shows no effect of either pre-saccadic predictive shifts or post-saccadic retinotopic persistence.**

|  | Distractor to right of target  (post-saccadic retinotopic persistence) | | Distractor to left of target  (pre-saccadic predictive remapping) | |
| --- | --- | --- | --- | --- |
|  | No. False-positives | Total trials | No. False-positives | Total trials |
| Pre-saccadic (200 to 0 ms before saccade offset) | | | | |
| Subject JV | 1 | 149 | 1 | 132 |
| Subject JS | 0 | 158 | 2 | 155 |
| Subject LV | 2 | 147 | 1 | 142 |
| Subject MK | 1 | 125 | 1 | 120 |
| Total | 4 | 579 | 5 | 549 |
| Post-saccadic (0 to 150 ms after saccade offset) | | | | |
| Subject JV | 1 | 68 | 1 | 67 |
| Subject JS | 0 | 66 | 0 | 87 |
| Subject LV | 0 | 66 | 0 | 69 |
| Subject MK | 0 | 55 | 3 | 68 |
| Total | 1 | 255 | 4 | 291 |
